# Nerve grafting for peripheral nerve injuries with extended defect sizes

**DOI:** 10.1007/s10354-018-0675-6

**Published:** 2018-12-13

**Authors:** Tim Kornfeld, Peter M. Vogt, Christine Radtke

**Affiliations:** 1 Department of Plastic, Aesthetic, Hand and Reconstructive Surgery, Hannover Medical School, Carl-Neuberg-Straße 1, 30625 Hannover, Germany; 2 Department of Plastic and Reconstructive Surgery, Medical University of Vienna, Währinger Gürtel 18–20, 1090 Vienna, Austria

**Keywords:** FDA, Cell transplantation, Artificial graft, Autograft, Allograft, FDA, Zelltransplantation, Kunsttransplantat, Autotransplantat, Allogenes Transplantat

## Abstract

Artificial and non-artificial nerve grafts are the gold standard in peripheral nerve reconstruction in cases with extensive loss of nerve tissue, particularly where a direct end-to-end suture or an autologous nerve graft is inauspicious. Different materials are marketed and approved by the US Food and Drug Administration (FDA) for peripheral nerve graft reconstruction. The most frequently used materials are collagen and poly(DL-lactide-ε-caprolactone). Only one human nerve allograft is listed for peripheral nerve reconstruction by the FDA. All marketed nerve grafts are able to demonstrate sufficient nerve regeneration over small distances not exceeding 3.0 cm. A key question in the field is whether nerve reconstruction on large defect lengths extending 4.0 cm or more is possible. This review gives a summary of current clinical and experimental approaches in peripheral nerve surgery using artificial and non-artificial nerve grafts in short and long distance nerve defects. Strategies to extend nerve graft lengths for long nerve defects, such as enhancing axonal regeneration, include the additional application of Schwann cells, mesenchymal stem cells or supporting co-factors like growth factors on defect sizes between 4.0 and 8.0 cm.

## Zusammenfassung

Der Goldstandard in der Versorgung von peripheren Nervendefekten ist nach wie vor die autologe Transplantation. Sollte eine Versorgung des Defekts mittels eines autologen Transplantats nicht möglich sein, so gibt es die Möglichkeit, auf von der U.S. Food and Drug Administration (FDA) zugelassene künstliche Nervenimplantate zurückzugreifen. Diese sind jedoch nur für periphere Nervendefekte <3,0 cm erprobt und zugelassen, für Defekte, welche diese 3,0 cm übersteigen, gibt es derzeit keine zugelassenen Alternativen. Für durch Tumorinfiltration oder Trauma entstehende Defekte werden diese aber dringend benötigt. Der Reviewartikel gibt einen Überblick über aktuelle Forschungsansätze mit dem Ziel der Transplantatversorgung langstreckiger Nervendefekte und zeigt die Notwendigkeit neuer, innovativer Forschungsansätze auch im Bereich der autologen Zelltransplantation.

## Introduction

Peripheral nerve damage is a severe and critical problem in all disciplines of surgery. With an overall incidence of 17.4% of iatrogenic acquired nerve lesions, this appears to be an underestimated major problem in patient treatment [1]. Several cases have been reported where a peripheral nerve was mistakenly used as a muscle tendon graft [2, 3] or nerve fibers have been damaged during anesthetic interventions [4–6]. The vast majority of peripheral nerve defects are caused by severe trauma or tumor infiltration. Noble et al. revealed that severe trauma leads to peripheral nerve injury with a prevalence of 2.8% in level 1 trauma patients [7].

Whether the nerve defect is caused iatrogenically or traumatically there is no difference in the surgical approach for nerve reconstruction. The current gold standard in nerve repair surgery is the tension free end-to-end suture. If this is not achievable an autologous nerve graft is indicated where a donor nerve is harvested and sutured to bridge the defect [8, 9]. The major disadvantage of this technique is the remarkable loss of sensitivity in the area of distribution and the limited availability of autologous donor tissue [10].

Artificial nerve grafts can be used as an alternative in cases of multiple nerve lesions where subsequent treatment with autografts is not possible due to limitation of donor tissue [11]. A variety of artificial nerve grafts is commercially available and approved by the FDA (US Food and Drug Administration) [12]. The data of the FDA revealed that collagen, chitosan and poly (DL-lactide-ε-caprolactone) are the most frequently used and approved materials (Table 1). Table 1 summarize all marketed and FDA-approved artificial nerve grafts for the surgical reconstruction of peripheral nerve tissue.

**Table 1:** FDA-approved nerve tubes for peripheral nerve repair. (Modified and supplemented from FDA Medical Device Database [104])

Name	Product name	Company	Date of approval	K510	Available Length (cm)	Material
Neuragen 3D	NeuraGen®	Integra Lifescience Corporation, Plainsboro, NJ, USA	Apr 24, 2014	K130557	3.0	Collagen
Neurogen Nerve Guide	NeuroGen®	Integra Lifescience Corporation, Plainsboro, NJ, USA	Jun 22, 2001	K011168	3.0	Collagen
Flexible collagen nerve	NeuroFlex®	Collagen Matrix Inc., Oakland, NJ, USA	Apr 03, 2014	K131541	2.5	Collagen
Collagen nerve cuff	NeuroMatrix®	Collagen Matrix Inc., Oakland, NJ, USA	Sep 21, 2001	K012814	n.a	Collagen
Reaxon Plus®	Reaxon Plus®	Medovent GmbH, Mainz, Germany	Dec 02, 2015	K143711	1.0	Chitosan
Neurotube®	Neurotube®	Neuroregen L.C.C, Bel Air, MD, USA	Mar 22, 1999	K983007	3.0	Poly(DL-lactide-ε-caprolactone)
Neurolac® Nerve Guide	Neurolac®	Polyganics BV, Rozenburglaan, Netherlands	Oct 20, 2011	K112267	2.0	Poly(DL-lactide-ε-caprolactone)
Neurolac® Nerve Guide	Neurolac®	Polyganics BV, Rozenburglaan, Netherlands	May 04, 2005	K050573	2.0	Poly(DL-lactide-ε-caprolactone)
Neurolac® Nerve Guide Models NG01-15/03, NG01-020/03, NG01-025/03, NG01-030/03	Neurolac®	Polyganics BV, Rozenburglaan, Netherlands	Oct 10, 2003	K032115	2.0	Poly(DL-lactide-ε-caprolactone)
AxoGen Avance®	AxoGen Avance®	AxoGen, Alachua, FL, USA	–	–	5.0	Human nerve allograft

The first collagen based peripheral nerve implants were approved for surgical intervention by the FDA in the early 2000s. Nearly simultaneously Neurogen® (Integra Lifescience Corporation, Plainsboro, NJ, USA) and Neuroflex® (Collagen Matrix Inc, Oakland, NJ, USA) have been introduced to the market. Neurogen® is a collagen based nerve tube demonstrating a satisfying recovery rate of 43% of level 1 trauma treated patients with peripheral nerve defects pending between 2.5–20.0 mm in length [13]. Neuoflex® is a collagen based nerve tube distributed by Stryker Corporation (Stryker Corporation, Kalamazoo, MI, USA). No clinical data is available after intensive literature research. Importantly, the collagen based materials have a significant low antigenicity and immunogenicity *in vivo* what makes them to a favorable material for *in vivo* applications [14].

Reaxon plus® (Medovent GmbH, Mainz, Germany) represents an approved conduit constructed out of the natural biomaterial chitosan. Chitosan was successfully used in rodent animal experiments for nerve regeneration over distances of up to 15.0 mm [15]. In this study nerve regeneration using chitosan nerve grafts was as effective as autologous nerve transplantation. Subsequent studies revealed that chitosan nerve grafts are able to reduce post-traumatic formation of neuroma and epineural fibrosis [16]. Unfortunately, chitosan was also reported to induce a foreign body reaction during degradation *in vivo* [17].

Neurotube® (Neuroregen L.C.C, Bel Air, MD, USA) and Neurolac® (Polyganics BV, Rozenburglaan, Netherlands) are nerve cuffs manufactured out of poly(DL-lactide-ε-caprolactone) and are approved for surgical reconstruction of nerve defects up to 3.0 cm in length. Neurotube® demonstrated successful facial nerve regeneration in several cases on defect sizes between 1.0 and 3.0 cm [18]. No signs of tissue rejection or inflammation processes occurred during application in human facial nerve repair. However, Duncan et al. report a case concerning the extrusion of a Neurotube® implant associated with inflammation processes [19]. Neurolac® is a competitive product to Neurotube® and one report suggests improved regeneration compared to autologous nerve grafts on a 1.0 cm peripheral nerve defect in rodents [20]. Supporting the results from den Dunnen et al. [19] a case series from Brazil indicates that the use of Neurolac® is a safe and successful procedure for peripheral nerve surgery [21].

AxoGen Avance® (AxoGen, Alachua, FL, USA) is the only FDA approved human nerve allograft. In several studies in animal models the efficiency of nerve regeneration through decellularized allografts was demonstrated, but was still inferior to isografts [22]. Clinical trials revealed that 87% of 132 nerve injuries treated with Axogen Avance® (AxoGen, Alachua, FL, USA) regained sensory and/or motor functions for treated peripheral nerve defect sizes between 5.0 and 50.0 mm [23]. Just recently Rinker et al. analyzed the results of the Ranger I study in regard to peripheral nerve defects on small diameter nerve defects on the hand [24]. Accordingly to the results of Brooks et al. [23] nerve regeneration with regained sensory function (S3+ or higher) was observable in 86% of the included cases.

All marketed and FDA approved nerve grafts demonstrate satisfying recovery on defect length from up to 3.0 cm with a minimal amount of side effects or regeneration failure. Despite these high number of FDA approved and commercially available artificial nerve grafts for reconstruction of peripheral nerve defects, no implant is approved and available for defect sizes extending 3.0 cm or longer in length. Nerve defects extending these 3.0 cm are usually considered to be critical [25]. Good clinical and non-clinical data is available for short nerve defects not extending 3.0 cm [13–21]. Recently Kaplan et al. criticized that only little clinical data is available for long gaped peripheral nerve defects and that rodent animals might not be a suitable animal model for translational research in peripheral nerve surgery [25]. Sufficient data concerning larger gap sizes is rare and the vast majority of scientific approaches does not lead to the expected results. Nevertheless surgical approaches for long gap nerve defects are needed during daily medical practice. Especially extended and multiple injuries of e. g. plexus nerves by trauma or long length tumor infiltration of peripheral nerve tissue are challenging in reconstruction These review gives a brief overview about current approaches in peripheral nerve surgery for critical gap sizes between 4.0 and 8.0 cm in small and large animal models using acellular nerve grafts or pre-seeded conduits.

## Material and methods

### Including criteria

Only scientific work in regard to peripheral nerve regeneration/reconstruction was included. Main criteria was a surgical nerve reconstruction in small/large animal models with artificial/non-artificial nerve grafts on nerve defect sizes ≥4.0 cm.

### Matches

In all, 30 original articles met the inclusion criteria of nerve reconstruction on nerve defects ≥4.0 cm. Four records were excluded after identified as duplicates. Two article were removed due to incomparable methods.

### Literature search

A literature search was performed via PubMed and Google Scholar. A key word search was performed using the following: “long gap nerve defects”, “extended nerve defects”, “reconstruction of extended nerve defects”, “critical sized nerve defects”, “nerve defects in large animal models”.

## Current experimental approaches in peripheral nerve reconstruction

### Small animal models

Small animal models are widely used in experimental *in vivo* investigation in the field of peripheral nerve surgery [22, 26–28]. High availability with moderate holding costs makes them a competitive and indispensable model for efficient and high throughput surgical testing experiments [29]. The sciatic nerves are easily accessible via a dorsal operation route. Postsurgical nerve regeneration can be evaluated *in vivo* either by electrophysiology, nerve pinch test or walking track analysis [30, 31]. Literature shows that the small animal models are also suitable for nerve surgery in settings with nerve defects extending 4.0 cm in length [32–40]. A disadvantage of the rodent model is that the dynamics of peripheral nerve regeneration may be different than in large animal models including humans, due to issues of scale [25, 41]. Table 2 gives a brief overview of current approaches in peripheral nerve surgery in small rodent animal models with or without cell transplantation and the addition of co-factors (Table 2).

**Table 2:** Small animal models in peripheral nerve surgery

	Defect size (cm)	Nerve	Animal	Implant	Time	Outcome	Quotation
*Small animal models in nerve surgery without cell transplantation*							
1	4.0	Tibial nerve	Rat	Chondroitinase ABC processed nerve	3 M	Higher numbers of regenerated axons compared to control	Neubauer et al. [32]
2	6.0	Sciaitic nerve	Rat	Allograft vs. autograft	20 W	Superior regeneration in allograft group compared to control	Saheb-al-Zamani et al. [33]
3	3.0, 5.0, 7.0	Saphenous nerve	Rabbit	Autograft	15 M	Decreasing regeneration with increasing defects size	Koller et al. [34]
4	5.0	Sciatic nerve	Rabbit	Muscle grafts	4 M	Light regeneration in muscle grafts	Mligiliche et al. [35]
5	10.0	Sciatic nerve	Rabbit	Muscle grafts	2 M	No regeneration	Hems et al. [36]
*Small animal models in nerve surgery with cell transplantation*							
6	4.0	Tibial nerve	Rabbit	Autologous vein with and without SC, Autograft as a control	2 M	Axonal regeneration in Isograft group and Vessel filled with Schwann cells	Zhang et al. [37]
7	1.0–6.0	Peroneal nerve	Rabbit	Vein	–	Regeneration on 3.0 cm. Poor outcome on long distances	Strauch et al. [39]
8	6.0	Peroneal nerve	Rabbit	Vein	4 M	Regeneration on full distance	Strauch et al. [38]
9	4.0	Ulnar nerve	Rat	ε-caprolactone-co-trimethylene carbonate filled with Schwann cells	12 M	No regeneration, extended formation of neuroma	Sinis et al. [40]

*M* month, *W* week

### Acellular nerve grafting

In cases were an alternative to the standard nerve end-to-end suture is desperately needed it can be defaulted to autologous nerve grafts or peripheral allografts as previously mentioned [11]. An acellular allograft or an artificial nerve graft appears as a widely investigated and suitable alternative. Especially isografts and acellular allograft have been extensively investigated in recent decades [42–44].

A recent study concerning nerve grafting with the previously mentioned acellular allograft was carried out on a 4.0 cm sciatic nerve defect in Fischer F344 rats with thermally decellularized allografts pretreated with chondroitinase ABC to breakdown axon inhibitory chondroitin sulfate proteoglycans (CSPGs) [32]. The authors report that nerve grafts pretreated with chondroitinase ABC shows a higher number of regenerated myelinated axons distal of the grafts suture compared to controls. Functionally they observed a higher number of positive nerve pinch tests and a reduction of retrograde axon growth in pretreated allografts indicating that chondroitinase ABC can support nerve regeneration over long nerve defect distances.

Whether the limited regeneration in long nerve grafts is caused by high doses of chondroitin sulfate proteoglycan and other scar related axon inhibitory elements or increasing number of senescent Schwann cells is not known. Saheb-Al-Zamani et al. investigated nerve regeneration through 2.0, 4.0 and 6.0 cm acellular nerve allografts [33]. Results demonstrate high expression of senescence markers (p16^INK4A^ and β‑galactosidase) in all grafts. Electron microscopy (EM) revealed a rising number of chromatin clumped cell nuclei with central involution in Schwann cells on acellular allografts >4.0 cm which is typical for cell aging. However, reorganization of chromatin in cell nuclei was not verifiable in short nerve grafts of 2.0 cm allograft and isografts respectively. Independent of this, nerve regeneration in autografts was superior to acellular allografts due to significantly higher numbers of myelinated fibers in distal parts of the nerve segments.

Another study investigated nerve regeneration using an autologous nerve graft on defect distances from 3.0, 5.0 and 7.0 cm in length in rabbit [34]. Maximum tetanic tension and number of myelinated axons deteriorate with increasing autograft length over a maximum 15-month observation time. Koller et al. reported that this result may indicate that nerve regeneration over a distance up to 7.0 cm is possible and the poor outcome regarding tetanic tension and remyelinization is explained by inferior vascularization in long length peripheral nerve grafts.

As an alternative to allo- and autografts, acellular muscle autografts have been investigated. The approach was first used in early the 1980s and 1990s [45–47]. Mligiliche et al. carried out a study using acellular muscle autografts on a 5.0 cm sciatic nerve defect in Japanese white rabbits [35]. After 4 months following implantation, compound action potentials (CMAP) and EM recordings were performed. Results indicate regeneration within the acellular muscle graft throughout the entire defect length with decreasing number of myelinated axons dependent upon graft length. CMAP shows light reinnervation on target muscle in each graft.

Hems et al. [36] performed a similar experiment in white New Zealand rabbits. Coapting a 5.0 and 10.0 cm sciatic nerve defect using cold freeze-dried muscle grafts compared to an autologous nerve graft. Light microscopy revealed inferior recovery in muscle grafts compared to controls. Nerve regeneration deteriorates after 2.0 cm and graft tissue was replaced by fat and connective tissue.

Data on long gaped peripheral nerve defects in small animals are limited possibly due to the issue of scale. However, small animal studies revealed that peripheral nerve regeneration through autologous donor nerves is nearly possible without side effects and that regeneration through artificial nerve grafts constructed from various materials is possible. Regeneration in allografts was reported to be less satisfactory than expected [32, 33]. Given these caveats in small animal models they have certainly provided valuable information that has advanced the field.

### Grafting with autologous donor cells

Schwann cells play a crucial role not only for myelination of peripheral nerve fibers, but for providing trophic support and structural guidance for axonal regeneration [48]. After nerve damage and during Wallerian degeneration when axons are degenerating, the Schwann cells disassociate from the axons and they begin to divide [49]. These changes are within the endoneural tubes or bands of Bügner. It is well established that Schwann cells not only form myelin for the electric insolation of peripheral nerves, but play a major role during the regeneration process of the peripheral nervous system by producing trophic factors and structural guidance for axonal growth [49, 50]. Several studies have investigated whether autologous Schwann cells can support peripheral nerve regeneration in artificial and non-artificial nerve grafts [51–55].

Experiments have been carried out to test Schwann cells on defect sizes <3 cm [53–56]. A 4.0 cm autologous monochanneled vein graft pre-seeded with 1 × 10^6^ autologous Schwann cells in rabbit was investigated by Zhang et al. [37]. Two months post-surgery electrophysiology showed evoked muscle potentials in both autologous controls and vein grafts-seeded with Schwann cells. Histological analysis revealed that the vein graft without cell suspension collapsed and the nerve formed a neuroma, whereas autologous controls showed almost normal full regenerated nerve tissue. The vein-Schwann cell grafts revealed adequate nerve fascicle formation and high numbers of myelinated axons in the distal part of the implant. Importantly, the autologous nerve graft seeded with Schwann cells demonstrated successful nerve regeneration over a distance of 4.0 cm. However, the results may be inferior to autologous nerve grafts.

Strauch et al. [38] performed a comparable experiment with some small variations in experimental settings on 6.0 cm nerve defects in rabbits. Previously, they were able to show that regeneration through 3.0 cm autologous vein grafts without cell transplantation is possible, but poor in longer grafts [39]. For this reason the experiments were repeated to investigate the effect of long autologous vein grafts seeded with 1 × 10^6^ Schwann cells in a peroneal nerve defect model. In contrast to the study by Zhang et al. [37] autologous vein grafts were harvested and filled with a mixture of matrigel and Schwann cells in suspension. Autologous veins filled only with matrigel were used as the control. As expected the controls filled with matrigel showed poor to no regeneration and a considerable amount of fibrosis. The autologous vein conduit filled with Schwann cells, however, showed myelinated axons in distal parts of the conduit.

In contrast to the previously described studies from Zhang et al. [37] and Strauch et al. [38] where a monochanneled vein autograft seeded with Schwann cells was successfully used on large nerve defect up to 6.0 cm, Sinis et al. [40] demonstrated negative results after treating a 4.0 cm nerve defect in rat, with a monochanneled ε‑caprolactone-co-trimethylene carbonate graft which was seeded with autologous Schwann cells. Autologous nerve graft controls demonstrate effective nerve regeneration as demonstrated by electrophysiology and immunohistology. Unfortunately, the newly developed ε‑caprolactone-co-trimethylene artificial nerve graft showed extended formation of neuroma in 13 out of 16 animals.

### Large animal models

Allograft and autografts in several nerve defect injury models in small animals have indicated that regeneration is possible on critical nerve defects. As previously mentioned Koller et al., Neubauer et al. and Saheb-Al-Zamani et al. were able to demonstrate that nerve regeneration in nerve defects up to a maximal length of 7.0 cm in small animals (rabbit, rat) is realistic [32–34].

In order to achieve results that are applicable to humans, a translational large animal model for peripheral nerve surgery is highly important. There is no standard large animal model for nerve repair studies, but a number of species are used in other disciplines. The ovine animal model is established as a standard surgery model regarding orthopedic questions to investigate treatment options in arthrosis, osteoarthritis or damage of cruciate ligaments [57–59]. Porcine animals are predominantly used in trauma and intensive care research [60–62] and feline models are used as ear, nose and throat (ENT) surgical standard models [63]. Artificial nerve conduits have been used successfully in different ovine animal models [64–66], yet there is no standard animal model on large peripheral nerve surgery. Table 3 gives an overview of study designs in peripheral nerve surgery using large animal models.

**Table 3:** Large animal models in peripheral nerve surgery

	Defect size (cm)	Nerve	Model	Implant	Time	Result	Author
*Large animal models in nerve surgery without cell transplantation or immunosuppression*							
1	8.0	Mesian nerve	Sheep	Autolog vs. allograft	6 and 10 M	No regeneration in allografts. Good results in autografts	Strasberg et al. [65]
2	8.0	Ulnar nerve	Swine	Autograft vs. allograft	6 and 10 M	Autograft significant superior to allograft	Atchabahian et al. [67]
3	7.0	Median nerve	Sheep	Autograft	6 and 9 M	Slightly decreased results in electrophysiology compared to untreated controls	Forden et al. [66]
4	5.0	Sciatic nerve	Cat	Freeze-dried alginate gel covered by polyglycolic acid mesh	7 M	Recovery through alginate gel is possible	Suzuki et al. [68]
5	5.0	Sciatic nerve	Cat	Freeze-dried alginate gel covered by polyglycolic acid mesh	10 M	Good regeneration without tubular structure	Sufan et al. [71]
6	8.0	Peroneal nerve	Dog	PGA-collagen-laminin	12 M	Regeneration throughout the conduit with some differences in histological appearance	Matsumoto et al. [72]
7	2.0, 5.0	Ulnar nerve	Primate	Maxon® collagen graft	14 M	Maxon® is superior compared to controls on defects sizes <2.0	Mackinson et al. [81]
8	6.0	Tibial nerve	Sheep	Vein filled with spider silk vs. autograft	10 M	Full functional recovery	Radtke et al. [64]
*Large animal models in nerve surgery with immunosuppression or cell transplantation/co-factors*							
9	4.0	Ulnar nerve	Primate	Allografts with MSC	6 M	Good recovery but inferior to isografts and Schwann cell seeded nerve grafts	Hu et al. [74]
10	8.0	Peroneal nerve	Sheep	Autograft, allograft + cyclosporine A	35 and 47 d	Major side effects due immunosuppression	Matsuyama et al. [99]
11	4.0	Ulnar nerve	Primate	Autograft, allograft + FK506	8 M	Comparable results of autograft and allograft + Fk506	Auba et al. [75]
12	5.0	Ulnar nerve	Primate	Autograft, allograft + anti-CD40 ligand	4 and 6 M	Anti-CD40 ligand monoclonal antibody can improve regeneration	Brenner et al. [73]
13	5.0	Ulnar nerve	Swine	Allografts + MHC Schwann cells + controls without cells	20 W	Good results regarding cold preservation of allografts	Brenner et al. [101]
14	5.0	Peroneal nerve	Dog	Allograft, autograft + bFGF	1 and 3 M	FGF can improve regeneration, 5.0 are possible without immunosuppression	Ide et al. [103]
15	8.0	Ulnar nerve	Swine	Autograft, allograft + FK506	24 W	FK506 improve regeneration, major side effects due to immunosuppression	Jensen et al. [102]

*M* month, *W* week, *d* day

In 1996 Strasberg et al. [65] used an ovine animal model to investigate an 8.0 cm peripheral nerve defect on median nerves surgically treated with either autologous nerve graft, nerve allograft, cold-preserved autograft or cold preserved allografts, thus introducing the ovine animal as a large animal model for peripheral nerve surgery. Analyses were performed after 6 and 10 months post-surgery. They concluded that both fresh autograft and cold-preserved autograft demonstrated nerve regeneration across the 8.0 cm nerve gap. Unfortunately, fresh allograft and cold preserved allografts demonstrated insufficient axonal regeneration.

Subsequently a study in the porcine animal model was performed with slightly modified parameters. In 12 adult swine an 8.0 cm bilateral ulnar nerve defect was induced and treated with an autologous nerve graft or a nerve allograft [67]. After 6 and 10 months nerves were harvested and histomorphometry revealed nerve regeneration and remyelinization in the autologous nerve transplant group. As previously shown in the ovine animal model [65] the regeneration of the allograft treated group remained unsatisfactory.

About 15 years later experiments in sheep were performed and reintroduced the sheep as a large animal model for nerve repair surgery [66]. Results from a median nerve defect in the sheep with a defect length of 5.0 cm coapted with a 7.0 cm autologous radial nerve graft was explored. After 6 and 9 months of surgery animals were euthanized and nerve grafts were analyzed regarding axonal regeneration. Electrophysiology revealed an insignificant difference between autograft and control. Results demonstrated regeneration throughout the entire graft length. Morphometric analyses showed axons with significantly smaller diameter in autologous nerve graft after 6 and 9 months compared to control, which is characteristic of regenerated axons. Extrafascicular and endoneural tissue were distinct. Forden et al. were able to show that the median nerve in sheep was an adequate model for peripheral nerve surgery. Thus, they were able to reproduce the results of Strasberg et al. [65] with an improved surgical and methodological approach.

Another study investigating freeze dried alginate gel on 5.0 cm sciatic nerve defects in cat [68]. Alginate was previously used in chronic wound healing and wound dressing [69, 70]. After 3 and 13 weeks compound muscle action potentials showed ongoing regeneration in the implant group with increasing latency and amplitudes after 13 weeks. Histomorphological analysis using electron microscopy (EM) demonstrated axon regeneration throughout the conduit 7 months following surgery. As already shown in the studies by Forden et al. [66] myelinated axons appeared with a significantly smaller diameter compared to controls.

In order to improve alginate as a material in peripheral nerve surgery, the experiments were repeated by Sufan et al. on a 5.0 cm sciatic defect model in feline animals two years later with a completely modified implant design [71]. A tubulated implant constructed from freeze dried alginate and polyglycolic acid (PGA) was compared to a non-tubulated alginate only graft. EM revealed regeneration in both graft groups independent of the tubulated and non-tubulated graft 10 months after surgery. As expected from prior studies from Suzuki et al. [68] and Forden et al. [66], axons appeared myelinated and smaller in diameter than in normal nerve tissue. Suzuki et al. were able to demonstrate that regeneration within the two different nerve grafts is comparable.

Following an entirely different approach Matsumoto et al. performed an experiment in 16 adult canine animals on an 8.0 cm peroneal nerve defect repaired with a polyglycolic based conduit filled with laminin coated collagen fibers [72]. The peroneal nerve was harvested 12 months post-surgery. Monthly recorded compound muscle action potentials (CMAPs) demonstrated incomplete regeneration throughout the 8.0 cm artificial nerve graft. EM showed that regeneration is still ongoing though decreasing axon diameter and number of myelinated axons. Immunohistochemistry could demonstrate growing neurofilament positive nerve fibers in the distal nerve stump. Matsumoto et al. were able to develop a suitable PGA-collagen implant for surgical intervention on large peripheral nerve defects.

Several experiments in nerve surgery have been carried out in non-human primates [73–75]. Primates are regarded as an important translational animal model in medicine for some situations [76, 77]. Nonetheless the cost and ethical concerns limit their use [78–80]. Mackinson et al. compared a synthetic glycolide trimethylene carbonate nerve graft with a collagen based conduit in a 2.0 cm and 5.0 cm ulnar/radial nerve defect in non-human primates [81]. The 14 month post-surgery results demonstrate a complete and excellent regeneration through the 2.0 cm grafts. Regeneration over a 5.0 cm nerve gap was significantly better in glycolide trimethylene carbonate conduits than in collagen based grafts but still poor in comparison to the 2.0 cm grafts.

Radtke et al. used a nerve implant constructed out of spider silk gained from the species *Nephilla *inside of a decellularized vessel for reconstruction of a 6.0 cm tibial nerve defect in ovine animals [64]. The spider silk nerve implant shows good results in electrophysiology and histological examination 10 months post-surgery. Results indicates that nerve regeneration through the spider silk nerve implant is at least as effective as autologous nerve grafts. The spider silk conduit was designed and prior to application in large animal model extensively tested *in vitro* and in small animal models *in vivo* [82–86]. Especially results of the current *in vitro* investigation of the spider silk based nerve implant emphasize that the implant might be suitable for nerve reconstruction on defect sides extending 6.0 cm in length [82]. Outcome of previous the *in vitro* study are currently being reevaluated in the ovine animal model [82]. Results of these *in vivo* investigation are pending. Although spider silk is a natural material it appears to be suitable for different surgical approaches due to its mechanical and thermal properties [87–93]. Radtke et al. briefly reviewed the use of silk in relation to peripheral nerve reconstruction and regeneration and discussed future material optimization and translation to daily medical practice [94].

### Grafting with cell transplantation on long gaped nerve defects

Mesenchymal stem cells (MSCs) play a major role in proliferation and differentiation during regenerating processes in tissue e. g. fat, bone and cartilage. An interesting property of MSCs with regard to nerve regeneration is the interaction they have with the innate and adaptive immune system as well as the down regulation of proinflammatory cytokines in damaged tissue [95].

Jun Hu et al. used this approach to investigate an allograft on a 4.0 cm ulnar nerve defect in non-human primates [74]. Allograft pre-seeded with MSCs were compared to Schwann cell seeded allograft, acellular autografts and acellular controls. After 6 months, the results indicated that regeneration in allografts with MSCs are better than in acellular controls, but still inferior to grafts pre-seeded with Schwann cells and isografts.

### Grafting with immunosuppression

Immunosuppression is needed to prevent foreign body reactions and rejections of implants [96]. Allografts have been reported to precipitate immune reactions resulting in implant rejection [97]. Cyclosporine A usually used after organ transplantation [98] was used to prevent allograft rejection in ovine animals on a 5.0 cm median nerve defect grafted with an 8.0 cm allograft [99]. Immunosuppression led to severe bacterial infections resulting in controlled experimental truncation. However, the results indicated that immunosuppression can prevent tissue rejection of allografts.

On the other hand, FK506 (tacrolimus) was able to demonstrate enhanced nerve regeneration in allografts in small rodents [100]. Auba et al. used a nonhuman primate model with immunosuppression [75]. A 4.0 cm nerve defect in nonhuman primate ulnar nerve was inducted and coapted with either allograft under immunosuppression (2 months only) with FK506 or isograft. Apart from slightly elevated nerve conduction velocities in the autologous control no significant difference was observed in the histological analysis 8 months after surgery. The investigators reported that this could indicate tissue rejection within the allograft.

Instead of using a systemic immunosuppression to prevent allograft rejection Brenner et al. used anti-CD 40 ligand monoclonal antibody on a 5.0 cm ulnar nerve defect in non-human primates [73]. To investigate the full influence of anti-CD 40 ligand monoclonal antibody a skin allograft was transplanted additionally to the nerve allograft in one group. Results demonstrated that anti-CD 40 ligand monoclonal antibody is able to avoid foreign body reactions that lead to tissue rejection ensuring equal regeneration compared to autografts. Skin grafts were rejected and indicate partial rejection of nerve allografts supposed to be caused by overwhelming foreign body reactions. A subsequent approach by Brenner et al. was to use pre-seeded allograft with MHC matched Schwann cells to downregulate foreign body reactions in porcine animals with and without pre-operative ultraviolet-B donor alloantigen injections [101]. Therefore a 5.0 cm ulnar nerve allograft model was designed. Allografts + MHC-Schwann cells resulted in excellent nerve regeneration compared to controls. Influence of UV-B donor alloantigen injections is not fully understood.

In 2005 Jensen et al. performed an experiment in 8 outbreed swine [102]. An 8.0 cm ulnar nerve defects was reconstructed with an autologous nerve graft and allografts. Six operated animals received an FK506 injection every 14 days to maintain immunosuppression. Three FK506 treated animals deceased before experimental endpoints suffering pulmonary abscess, infarcted bowel or cardiac arrest. Results demonstrate a comparable regeneration in FK506 allografts compared to untreated autografts. Autografts from animals treated with FK506 showed a slight improved regeneration compared to autologous controls.

### Grafting without immunosuppression

Brenner et al. [73, 101], Matsuyama et al. [99] and several other investigators demonstrated that allografting without immunosuppression leads to tissue rejection. Ide et al. tested an acellular allograft in combination with fibroblast growth factor (FGF) on a 5.0 cm peroneal nerve defect in a canine animal model [103]. They report that nerve regeneration throughout the allograft is possible without immunosuppression. However, the regeneration is less than that obtained from autografts. Clearly the use of immunosuppression is an important issue for use of autografts and research in this area is critical for non-autologous biosynthetic graft construction and use in clinical studies for nerve repair.

## Conclusion

### Translation to clinical practice

Ongoing developments in peripheral nerve surgery are auspicious. Allografts are nearly a perfect alternative to the current gold standard technique, especially because immunosuppression is not indicated anymore to avoid a foreign body reaction. Nerve grafting with cell suspensions e. g. with Schwann cells shows promising results on small defect sizes in animals but is limited on translation to human organism by highly regulated local laws for transplantation of human stem cells. Muscle grafts and mono channeled nerve grafts are not appropriate for reconstruction of long gaped peripheral nerve defects. Nerve reconstruction with biomaterials like spider silk are as good as the autologous gold standard. Research is still ongoing and transfer to the human organism is pending.

### Research approaches

A literature search demonstrated that scientific approaches and animal models in peripheral nerve surgery are extremely versatile with the result of limited and impeded possibilities of comparison. Scientists in the field of peripheral nerve surgery should focus on comparable approaches regarding animal models and methods to produce comparable and competitive results. Nevertheless most reviewed approaches are promising regarding future clinical translation. Especially the transplantation of autologous donor cells and factors within artificial and non-artificial nerve grafts might be advantageous in future settings.

### Effect on daily medical practice

The autologous nerve graft is still and with good reason the gold standard for reconstruction of peripheral nerve tissue. Nevertheless autologous donor material is highly limited in number and often mismatch the nerve defects of the reconstruction side. Especially multiple long gaped plexus injuries are challenging during reconstruction. Furthermore the donor side morbidity after autograft harvest can lead to post-operative complications e. g. with neuroma formation, permanent sensitivity loss and adherent scars. Obviously autologous donor harvest is limited. In those surgical settings with limited availability of donor tissue the use of artificial nerve grafts should be recommended for peripheral nerve defects <3.0 cm. Both artificial nerve graft e. g. NeuroGen®, Neurotube®, Neurolac® and nerve allografts e. g. AxoGen® Avance can be recommended as alternative approaches for nerve reconstruction after literature analysis. Translational and alternative approaches for the reconstruction of long gaped defect sides are desperately needed. Especially the cell free approaches including allografts or spider silk nerve implants could be a contemporary and sufficient transfer technology beside neurotization until cell based procedures are safe and a statutory basis is set.

## Abbreviations

CMAP: Compound action potentials
CSPGs: Chondroitin sulfate proteoglycans
EM: Electron microscopy
ENT: Ear, nose and throat
FDA: US Food and Drug Administration
FGF: Fibroblast growth factor
MHC: Major histocompatibility complex
MSCs: Mesenchymal stem cells
PGA: Polyglycolic acid
